# Critical weight loss predicts poor prognosis in nasopharyngeal carcinoma

**DOI:** 10.1186/s12885-016-2214-4

**Published:** 2016-02-29

**Authors:** Qi Zeng, Lu-Jun Shen, Xiang Guo, Xin-Ming Guo, Chao-Nan Qian, Pei-Hong Wu

**Affiliations:** State Key Laboratory of Oncology in South China; Collaborative Innovation Center for Cancer Medicine, Guangzhou, 510060 China; Department of Medical Imaging and Interventional Oncology, Sun Yat-sen University Cancer Center, 651 Dongfeng Road East, Guangzhou, 510060 PR China; Department of Nasopharyngeal Carcinoma, Sun Yat-sen University Cancer Center, Guangzhou, PR China; Department of Pharmacy, the Fifth Affiliated Hospital, Sun Yat-sen University, Zhuhai, 519000 PR China

**Keywords:** Weight loss, Nasopharyngeal carcinoma, Radiotherapy, Survival

## Abstract

**Background:**

The impacts of weight loss on prognosis in nasopharyngeal carcinoma (NPC) remain unclear. The present study was therefore undertaken to investigate the association between critical weight loss and long-term survival in NPC patients.

**Methods:**

The eligible 2399 NPC patients were reviewed. Weight change was categorized into critical weight loss (CWL) and non-critical weight loss (Non-CWL). The associations of CWL with long-term survival were analyzed by Cox regression in the entire patient and two subsets. Propensity score matching was performed to reduce the effects of confounding factors.

**Results:**

CWL was defined as body weight loss of ≥4.6 %. Compared with patients without CWL, patients with CWL had significantly lower 5-year OS (72.4 vs. 79.3 %, *P* < 0.001), FFS (71.1 vs. 78.4 %, *P* <0.001), and LR-FFS (78.1 vs. 84.8 %, *P* <0.001), respectively. After adjustment for potential confounders, CWL remained an independence prognostic factor for OS (*HR* = 1.352; 95 % CI 1.160–1.576; *P* < 0.001), FFS (*HR* = 3.275; 95 % CI 1.101–9.740; *P* = 0.033), and LR-FFS (*HR* = 6.620; 95 % CI 2.990–14.658; *P* < 0.001), respectively. Furthermore, subgroup analysis in the cohort of patients received concurrent chemoradiotherapy or radiotherapy alone confirmed the results in the entire patient even after the propensity-score matching. In IMRT cohort, CWL was also significantly associated with a lower OS (*P* = 0.04) and FFS (*P* = 0.04).

**Conclusions:**

CWL has a significant and independent impact on long-term survival in nasopharyngeal carcinoma patients.

**Electronic supplementary material:**

The online version of this article (doi:10.1186/s12885-016-2214-4) contains supplementary material, which is available to authorized users.

## Background

Body weight loss (WL) during radiotherapy is a frequently observed problem among patients with head and neck cancer (HNC) [1–4], but there have been controversies over the impact of weight loss during radiotherapy on survival. In the study of Pai et al. [5], comparing with patients with less WL, patients with greater WL during radiotherapy have significantly worse survival in patients with higher pre-radiotherapy body mass index (BMI). Two 2013 studies found weight loss is an independent prognostic factor for disease-free survival, but not for overall survival [6, 7]. Nasopharyngeal carcinoma (NPC) is a distinct form of HNC due to unique clinical, etiological and biological characteristics [8–10]. Shen et al. [11] reported high weight loss is independently associated with poor survival in NPC patients with lower BMI. To date, the impact of weight loss on long-term survival in NPC patients remain unclear, given the diversity of chemotherapy regimen and radiotherapy technique.

In the present study, we used data obtained from a large database of NPC patients in our institute to investigate the association between weight loss and long-term survival in the entire patient and its two subsets. Propensity score matching was performed to reduce the effects of confounding factors.

## Methods

### Study cohort

From a cohort of newly diagnosed nasopharyngeal carcinoma patients between January 2001 and January 2005, this study was approved by the ethics committee of Sun Yat-sen University Cancer Center. This was a retrospective analysis of routine data and therefore we were granted a waiver of individual informed consent from the ethics committee of Sun Yat-sen University. The data were collected by trained interviewers and analyzed anonymously. Patients who met the following criteria were selected: (i) Histologically confirmed nonkeratinizing or undifferentiated NPC (World Health Organization type II or III); (ii) patients newly diagnosed without evidence of systemic metastasis; (iii) KPS (Karnofsky performance scale) score ≥80; (iv) Completion of the scheduled total radiotherapy dose. The exclusion criteria included: lack of complete weight measurement at baseline and/or at the end of radiotherapy. The final study cohort was composed of 2399 patients. All patients were evaluated by the following examinations before treatment: complete patient history, physical examination, CT or MRI of the neck and nasopharynx, chest radiography, abdominal sonography, and acquisition of whole body bone scans by single photon emission computed tomography (ECT).

### Data collection

Medical records were reviewed to extract data on patient and tumor characteristics, including age, gender, the sixth edition of Union for International Cancer Control /American Joint Committee on Cancer (UICC/AJCC) stage [12], radiotherapy techniques and dose, treatment group (radiotherapy or combined chemo-radiotherapy), BMI [defined as pre-radiotherapy weight (kg) divided by the square of height (meter)], smoking status at diagnosis, categorized into two groups: (i) never-smokers referred to patients who never smoke; (ii) ex-smokers referred to former smokers who had stopped smoking and smokers who smoking until the day of hospitalization. Pre-radiotherapy body weight was measured within 7 days before radiotherapy (RT), and post-radiation body weight was measured within 7 days after completion of RT. Weight loss was based on the equation (Pre-radiotherapy weight –Post-radiotherapy weight)/ Pre-radiotherapy weight × 100 %.

### Treatment

Radiotherapy techniques included two-dimensional conventional radiotherapy, which included X-ray simulation (*n* = 1897) and CT simulation (*n* = 315) for radiotherapy treatment planning, three-dimensional conformal radiotherapy (3D-CRT, *n* = 49), intensity-modulated radiotherapy (IMRT, *n* = 138). These details have been previously described by Shen et al.[11]. Briefly, Conventional radiation therapy was performed by 2 Gy per fraction with five daily fractions per week up to a total dose of 68–78 Gy. For 3D-CRT, the total prescribed dose was 66–72 Gy to the gross tumor volume of nasopharynx (GTVnx), 60 to 70 Gy to the region involved by the metastatic lymph nodes (GTVnd). For IMRT, the prescription dose was 68 Gy to GTVnx, 60 to 64 Gy to GTVnd. Combined modality therapy for most locoregionally advanced NPC included induction chemotherapy followed by concurrent chemoradiotherapy (*n* = 184), concurrent chemoradiotherapy (*n* = 306), induction chemotherapy (*n* = 494), induction chemotherapy or concurrent chemoradiotherapy plus adjuvant chemotherapy (*n* = 50), and miss data (*n* = 299). The induction or adjuvant chemotherapy regimen was mainly cisplatin plus fluorouracil (5-Fu), with cisplatin (70 to 100 mg/m^2^) given on Day 1 and 5-fluorouracil (500 to 750 mg/m^2^) on Days 1–5, repeated every 3–4 weeks, for 2 to 3 cycles. The concurrent chemotherapy regimen was mainly cisplatin alone, with cisplatin (30–40 mg/m^2^ on Day 1) given intravenously weekly for 5–7 weeks or cisplatin (80–100 mg/m^2^) given intravenously 3-weekly for three cycles.

### Follow-up and end points

The primary endpoint was overall survival rates (OS), the secondary endpoints were failure-free survival rates (FFS), locoregional failure-free survival rates (LR-FFS), and distant failure-free survival rates (D-FFS). OS was defined as the length of time from the date of beginning therapy to the date of death from any cause. FFS was defined as the time between the date of beginning therapy and the date of treatment failure or death from any cause, whichever was first. LR-FFS was defined as the time to first recurrence at the nasopharyngeal region and/or in the cervical region after radiotherapy, not including salvage procedures. D-FFS was defined as the time from the date of beginning therapy to the first distant failure. The last follow-up visit occurred in August 2011.

### Statistical analysis

Categorical variables were compared with *χ*^2^ tests (or Fisher’s exact test, if indicated) and continuous variables with Student’s t test. Receiver operating characteristic curve (ROC) analysis was used to select the cutoff point of weight loss. Survival analysis was carried out using the Kaplan–Meier method and compared with the log-rank test. Multivariate analyses with the Cox proportional hazards model were used to test for independent significance by backward elimination of insignificant explanatory variables of the different parameters. The Cox proportional hazards model was also used to calculate the hazard ratio (HR). The interaction between weight loss and BMI was assessed using Cox regression. The statistical analyses were performed using SPSS version 19.0 (SPSS, Inc., an IBM Company, Chicago, IL, USA). A two-sided *P*-value <0.05 was taken as statistically significant. Given the differences in the baseline characteristics between critical weight loss and non-critical weight loss groups, propensity-score matching in R Statistical Software (version 3.1.3; R Foundation for Statistical Computing, Vienna, Austria) was performed using the MatchIt package with nearest-neighbor 1-to-1 matching [13].

## Results

### Demographic, patterns of treatment failure, and survival

A total of 2399 nasopharyngeal carcinoma patients were included in this study, with a median age of 46 years (range, 13–78 years). The ratio of male to female was 3.19:1, with 1826 males and 573 females. The sixth edition of the UICC/AJCC clinical stage distribution was: stage I, 126 (5.3 %); stage IIa, 23 (1.0 %); stage IIb, 816 (34.0 %); stage III, 971 (40.5 %), and stage IVa 377 (15.7 %); and stage IVb 86 (3.6 %). Overall, 1066 (44.4 %) patients were treated with radiotherapy (RT) alone and 1333 (55.6 %) received combined chemo-radiotherapy (CRT). The median follow-up for the whole group was 85.3 months (range: 1.6–124.7 months), for alive patients was 93.6 months (range:74.2–124.7 months). 546 (22.8 %) patients developed locoregional relapse, 158 (6.6 %) developed distant metastases, and 729 (30.4 %) died. The 3- and 5-year survival rates were as follows: OS, 84.2 and 75.4 %; FFS, 82.9 and 74.3 %; LR-FFS, 87.7 and 81.1 %; and D-FFS, 95.7 and 94.2 %.

### Determination of cutoff points for weight loss and the distribution of patients characteristics in the entire patient cohort

Because OS was the primary endpoint in this study, the cutoff point for OS was selected as the optimal cutoff value using ROCanalysis. The result indicated the cutoff value of weight loss was 4.6 % (the sensitivity was 62.7 % and the specificity was 46.9 %) with an area of 0.546 (95 % CI, 0.521–0.572; *P* <0.001). Critical weight loss (CWL) was defined as body weight loss of ≥4.6 %. CWL was observed in 56.0 % (1343/2399) of patients. Mean weight loss was 9.1 (±3.6) %. In patients without critical weight loss, 656 patients (62.1 %) had <4.6 % weight loss, 152 patients (14.4 %) had no weight loss, and 248 patients (23.5 %) had weight gain. As shown in Table 1, there were no differences in the distribution of gender, smoking status or radiotherapy dose for the entire patient cohort when categorized by cut-off points. However, significant differences were observed in terms of age, clinical stage, T-stage, N-stage, treatment group, and BMI. Older patients and higher BMI were more frequent in patients with CWL. In addition, patients without critical weight loss exhibited more patients with advanced T-stage, N-stage, or clinical stage. Accordingly, the proportion of patients received combined chemoradiotherapy was higher in the non-critical weight loss group.

**Table 1:** Baseline characteristics of nasopharyngeal carcinoma patients with and without critical weight loss

Characteristics	Non-critical weight loss (*N* = 1056)	Critical weight loss (*N* = 1343)	*P*
Age (y) media (range)	45 (13–78)	46 (13–78)	0.004
Gender (%)			
Male	817 (77.4)	1009 (75.1)	0.202
Female	239 (22.6)	334 (24.9)	
Clinical stage (%)			
I-II	476 (45.1)	489 (36.4)	<0.001
III-IV	580 (54.9)	854 (63.6)	
T-stage (%)			
T1-2	648 (61.4)	745 (55.5)	0.004
T3-4	408 (38.6)	598 (44.5)	
N-stage (%)			
N0-1	744 (70.5)	841 (62.6)	<0.001
N2-3	312 (29.5)	502 (37.4)	
Treatment group (%)			
RT	547 (51.8)	519 (38.6)	<0.001
CRT	509 (48.2)	824 (61.4)	
Smoking status (%)			
never-smokers	552 (52.3)	697 (51.9)	0.856
ex-smokers	504 (47.7)	646 (48.1)	
RT dose (Gy), media (range)	70 (60–87)	70 (60–86)	0.127
BMI (kg/m2), media (range)	22.04 (14.04–35.36)	22.77 (13.61–39.06)	<0.001

*Notes*: critical weight loss: weight loss ≥ 4.6 %; *RT* Radiotherapy alone, *CRT* Combined chemo-radiotherapy, *BMI* Pre-RT weight (kg) divided by the square of height (meter)

### Impact of critical weight loss on survival in the entire patient

**Fig. 1:**
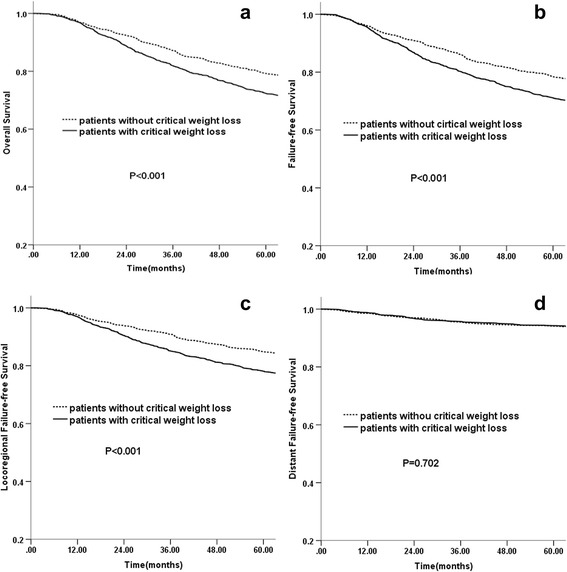
Comparison of survival between patients with and without CWL

Compared with patients without CWL, patients with CWL had significantly lower 5-year OS (72.4 vs. 79.3 %, *P* < 0.001; Fig. 1a), FFS (71.1 vs. 78.4 %, *P* <0.001; Fig. 1b), and LR-FFS (78.1 vs. 84.8 %, *P* <0.001; Fig. 1c), respectively. No significant benefit was observed for D-FFS (94.3 vs. 94.1 %, *P* =0.702; Fig. 1d) between the two groups. The unadjusted Cox regression analysis (Table 2) showed that critical weight loss was significantly associated with a worse OS (*HR* = 1.411; 95 % CI 1.214–1.639; *P* < 0.001), FFS (*HR* = 1.383; 95 % CI 1.193–1.603; *P* < 0.001), and LR-FFS (*HR* = 1.487; 95 % CI 1.248–1.771; *P* < 0.001). After adjustment for age (continuous variable), gender (female vs. male), T stage (T1-2 vs. T3-4), N stage (N0-1 vs. N2-3), treatment group (RT vs. CRT), BMI (continuous variable), smoking status (never smokers vs. ex-smokers), radiotherapy dose (continuous variable), and (weight loss) × BMI, critical weight loss remained an independent prognostic factor for OS (*HR* = 1.352; 95 % CI 1.160–1.576; *P* < 0.001), FFS (*HR* = 3.275; 95 % CI 1.101–9.740; *P* = 0.033), and LR-FFS (*HR* = 6.620; 95 % CI 2.990–14.658; *P* < 0.001). There were no interactions between weight loss and BMI for OS (*P* = 0.119), FFS (*P* = 0.099), D-FFS (*P* = 0.993). For LR-FSS, the (weight loss) × BMI interaction term was significant (*P* < 0.001).

**Table 2:** Cox regression analyses of the association between critical weight loss and survival in the entire patient cohort and its two subsets

	OS		FFS		LR-FFS		D-FFS	
	HR (95 % CI)	*P*	HR (95 % CI)	*P*	HR (95 % CI)	*P*	HR (95 % CI)	*P*
Patient cohort (*n* = 2399)								
Unadjusted model	1.411 (1.214–1.639)	<0.001	1.383 (1.193–1.603)	<0.001	1.487 (1.248–1.771)	<0.001	0.941 (0.688–1.287)	0.702
Adjusted model	1.352 (1.160–1.576)	<0.001	3.275 (1.101–9.740)	0.033	6.620 (2.990–14.658)	<0.001	1.432 (0.149–13.736)	0.755
Subset I (*n* = 1374)								
Matched/Unadjusted model	1.554 (1.259–1.917)	<0.001	1.539 1.251–1.895)	<0.001	1.577 (1.241–2.004)	<0.001	1.276 (0.763–2.134)	0.352
Matched/Adjusted model	1.515 (1.227–1.871)	<0.001	1.504 (1.221–1.852)	<0.001	9.395 (2.965–29.765)	<0.001	0.846 (0.02–36.683)	0.931
Subset II (*n* = 110)								
Matched/Unadjusted model	4.857 (1.049–22.483)	0.043	4.857 (1.049–22.483)	0.043	5.143 (0.601–44.027)	0.135	2.037 (0.185–22.470)	0.560
Matched/Adjusted model	4.998 (1.080–23.141)	0.040	4.986 (1.077–23.086)	0.040	5.356 (0.623–46.011)	0.126	1.656 (0.144–19.117)	0.680

*Notes*: Patient cohort: the entire patients; Subset I: the patient cohort received radiotherapy alone or concurrent chemoradiotherapy after matching. Subset II: The patient cohort received IMRT after matching. Critical weight loss: weight loss ≥4.6 %. Adjusted for age (continuous variable), gender (female vs. male), UICC T stage (T1-2 vs. T3-4), UICC N stage (N0-1 vs. N2-3), treatment group (RT vs. CRT), BMI (continuous variable), smoking status (Never smokers vs. ex-smokers), radiotherapy dose (continuous variable)

### Impact of critical weight loss on survival in the patients received concurrent chemoradiotherapy or radiotherapy alone

**Fig. 2:**
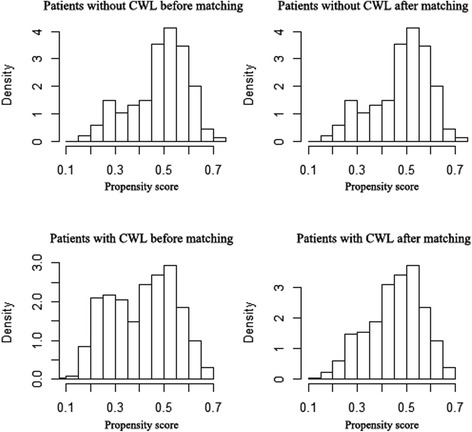
Histograms of propensity scores before and after matching in patients received radiotherapy alone or concurrent chemoradiotherapy CWL: critical weight loss (weight loss ≥4.6 %)

The modes of chemotherapy in our study varied differently, which might have a confounding effect. Patients received induction chemotherapy alone and adjuvant chemotherapy was excluded. We developed a new cohort to analyze the impact of CWL on survival, in which patients received radiotherapy alone (*n* = 1066), concurrent chemoradiotherapy alone (*n* = 306) or induction chemotherapy plus concurrent chemoradiotherapy (*n* = 184). The characteristics of the patient cohort were summarized in Table 3. The propensity-score matching was performed to reduce the differences in the baseline characteristics, matching variables included age, clinical stage, T-stage, N-stage, treatment group, radiotherapy dose, BMI. After matching, baseline characteristics were similar in the two groups (Table 3). Figure 2 shows the histograms before and after matching. The histograms before matching on the left differ to a great degree. The histograms after matching on the right are very similar.

**Table 3:** Characteristics stratified by critical weight loss before and after propensity-score matching in patients received radiotherapy alone or concurrent chemoradiotherapy

	Before Matching			After Matching		
Characteristics	Non-CWL (*N* = 687)	CWL (N = 869)	*P*	Non-CWL (*N* = 687)	CWL (*N* = 687)	*P*
Age (y) media (range)	45 (13–78)	47 (14–77)	0.005	45 (13–78)	46 (15–77)	0.050
Sex (%)						
Male	522 (76.0)	657 (75.6)	0.863	522 (76.0)	518 (75.4)	0.801
Female	165 (24.0)	212 (24.4)		165 (24.0)	169 (24.6)	
Clinical stage (%)						
I-II	388 (56.5)	381 (43.8)	<0.001	388 (56.5)	352 (51.2)	0.051
III–IV	299 (43.5)	488 (56.2)		299 (43.5)	335 (48.8)	
T-stage (%)						
T1-2	481 (70.0)	529 (60.9)	<0.001	481 (70.0)	458 (66.7)	0.182
T3-4	206 (30.0)	340 (39.1)		206 (30.0)	229 (33.3)	
N-stage (%)						
N0-1	536 (78.0)	592 (68.1)	<0.001	536 (78.0)	521 (75.8)	0.337
N2-3	151 (22.0)	277 (31.9)		151 (22.0)	166 (24.2)	
Treatment group (%)						
RT	547 (79.6)	519 (59.7)	<0.001	547 (79.6)	509 (74.1)	0.015
CRT	140 (20.4)	350 (40.3)		140 (20.4)	178 (25.9)	
Smoking status (%)						
never-smokers	375 (54.6)	450 (51.8)	0.272	375 (54.6)	365 (53.1)	0.588
ex-smokers	312 (45.4)	419 (48.2)		312 (45.4)	322 (46.9)	
RT dose (Gy), media (range)	70 (60–87)	70 (60–86)	0.081	70 (60–87)	70 (60–86)	0.599
BMI (kg/m2) media (range)	22.05 (14.04–34.89)	22.83 (15.35–39.06)	<0.001	22.05 (14.04–34.89)	22.41 (15.35–32.99)	0.058

*Notes*: *RT* Radiotherapy alone, *CRT* Combined chemo-radiotherapy, *BMI* Pre-RT weight (kg) divided by the square of height (meter)

The unadjusted Cox regression analysis after propensity score matching showed that CWL (table 2) was significantly associated with a worse OS (*HR* = 1.554; 95 % CI 1.259–1.917; *P* < 0.001), FFS (*HR* = 1.539; 95 % CI 1.251–1.895; *P* < 0.001), and LR-FFS (*HR* = 1.577; 95 % CI 1.241–2.004; *P* < 0.001). After adjustment for age (continuous variable), gender (female vs. male), T stage (T1-2 vs. T3-4), N stage (N0-1 vs. N2-3), treatment group (RT vs. CRT), BMI (continuous variable), smoking status (never smokers vs. ex-smokers), radiotherapy dose (continuous variable), CWL remained an independent prognostic factor for OS (*HR* = 1.515; 95 % CI 1.227–1.871; *P* < 0.001), FFS (*HR* = 1.504; 95 % CI 1.221–1.852; *P* < 0.001), and LR-FFS (*HR* = 9.395; 95 % CI 2.965–29.765; *P* < 0.001).

### Impact of critical weight loss on survival in IMRT cohort

There were 138 patients received IMRT in the study. Further analysis was performed in IMRT cohort. Table 4 showed the characteristics of the patients before and after propensity score matching. Total fifty-five pairs were confirmed and baseline characteristics were similar in the two groups after matching. The multivariate analysis was performed in the IMRT cohort with the covariates, including age (continuous variable), gender (female vs. male), T stage (T1-2 vs. T3-4), N stage (N0-1 vs. N2-3), treatment group (RT vs. CRT), BMI (continuous variable), smoking status (never smokers vs. ex-smokers), radiotherapy dose (continuous variable), Patients with CWL had an HR of death of 4.998 (95 % CI, 1.080-23.141; *P* = 0.040), HR of disease failure of 4.986 (95 % CI, 1.077–23.086; *P* = 0.040) compared with patients without CWL. But CWL wasn’t significantly associate with LR-FFS (*P* = 0.126) and D-FFS (*P* = 0.680).

**Table 4:** Characteristics stratified by critical weight loss before and after propensity-score matching in patients received IMRT

	Before Matching			After Matching		
Characteristics	Non-CWL (*N* = 55)	CWL (*N* = 83)	*P*	Non-CWL (*N* = 55)	CWL (*N* = 55)	*P*
Age (y) media (range)	41 (18–60)	42 (13–73)	0.538	41 (18–60)	40 (15–67)	0.832
Sex (%)						
Male	39 (70.9)	63 (75.9)	0.513	39 (70.9)	41 (74.5)	0.669
Female	16 (29.1)	20 (24.1)		16 (29.1)	14 (25.5)	
Clinical stage (%)						
I-II	24 (43.6)	20 (24.1)	0.016	24 (43.6)	19 (34.5)	0.329
III-IV	31 (56.4)	63 (75.9)		31 (56.4)	36 (65.5)	
T-stage (%)						
T1-2	30 (54.5)	36 (43.4)	0.198	30 (54.5)	24 (43.6)	0.252
T3-4	25 (45.5)	47 (56.6)		25 (45.5)	31 (56.4)	
N-stage (%)						
N0-1	44 (80.0)	40 (48.2)	<0.001	44 (80.0)	38 (69.1)	0.189
N2-3	11 (20.0)	43 (51.8)		11 (20.0)	17 (30.9)	
Treatment group (%)						
RT	34 (61.8)	24 (28.9)	<0.001	34 (61.8)	23 (41.8)	0.036
CRT	21 (38.2)	59 (71.1)		21 (38.2)	32 (58.2)	
Smoking status (%)						
Never-smokers	33 (60.0)	48 (57.8)	0.800	33 (60.0)	31 (56.4)	0.699
Ex-smokers	22 (40.0)	35 (42.2)		22 (40.0)	24 (43.6)	
RTdose (Gy), media (range)	68 (66–81)	68 (66–68)	0.289	68 (66–81)	68 (66–88)	0.254
BMI (kg/m2), media (range)	23.34 (14.86–30.08)	23.23 (15.35–30.82)	0.565	23.34 (14.86–30.08)	23.23 (15.35–30.82)	0.268

*Abbreviations*: *RT* Radiotherapy alone, *CRT* Combined chemo-radiotherapy, *BMI* Pre-RT weight (kg) divided by the square of height (meter)

### Revalidation of the impact of weight loss on survival by another threshold of critical weight loss

To further clarify the impact of weight loss on survival, a recommended threshold of CWL (≥5 %) by the American Society for Parenteral and Enteral Nutrition was used, and the entire patient was divided into three categories: patients with ≥5 % weight loss (*n* = 1277); patients with <5 % weight loss (*n* = 722); patients with weight gain and without weight loss (*n* = 400). As showed in Additional file 1: Figure S1, No significant benefit was observed for 5-year OS (79.5 vs. 77.5 %, *P* =0.401) between patients with <5 % weight loss and patients with weight gain and without weight loss (*n* = 400). However, compared with the above two categories, patients with ≥5 % weight loss had significantly lower 5-year OS (72.4 %, *P* < 0.05). These results confirmed our conclusions.

## Discussion

Weight loss is common among HN cancer patients, especially for those with advanced tumor stage, or a higher body mass index before treatment, or the use of concurrent chemotherapy [3, 14]. Several different definitions were used to define critical / high weight loss or severe malnutrition [2, 6, 11, 15, 16]. We defined critical weight loss as body weight loss of ≥4.6 %, based on the result of ROC analysis for OS in the entire patient, because OS was the primary endpoint in this study. The ratio of critical weight loss in HNC patients was reported to vary from 19 % to 60 % [3, 11, 17, 18], in the present study, 56.0 % (1343/2399) patients developed CWL. Although over half of patients presented with CWL during radiotherapy, there was limited information regarding the association between CWL and long-term survival. The aim of the present study is to elucidate the impact of CWL on survival in NPC patients and provide new clues for clinical intervention to improve their survival.

In our study, after adjustment for all the potential confounding factors, patients with CWL had an HR of death of 1.352 (95%CI 1.160–1.576; *P* < 0.001), HR of disease failure of 3.275 (95 % CI, 95 %CI 1.101–9.740; *P* = 0.033), and HR of locoregional recurrence of 6.620 (95%CI 2.990–14.658; *P* < 0.001) compared with patients without critical weight loss. The WL × BMI interaction term was significant (*P* < 0.001) only for LR-FSS, indicating that the prognostic impact of weight loss differed significantly on the basis of BMI. Furthermore, given the diversity of chemotherapy modality and radiotherapy technique, we developed two additional subsets to confirm the results. In addition, regression analysis cannot reliably adjust for differences in covariates when there are substantial differences in the distribution of these covariates between two groups. When regression approaches cannot remove all or nearly all the bias, alternative strategies such as propensity score matching can be used [19]. In the cohort of patients received concurrent chemoradiotherapy and radiotherapy, excluding the interference of induction chemotherapy alone and adjuvant chemotherapy, CWL remain an independent prognostic factor for OS, FFS, LR-FFS even after propensity score matching. In addition, IMRT has been shown to increase the locoregional control probability while decreasing the complication rate [20, 21], in the IMRT cohort of our study, Patients with CWL had an HR of death of 4.998 (95 % CI, 1.080–23.141; *P* = 0.040), HR of disease failure of 4.986 (95 % CI, 1.077–23.086; *P* = 0.040) compared with patients without CWL. But CWL wasn’t significantly associate with LR-FFS (*P* = 0.126) in IMRT cohort. It is likely that the sample size (*n* = 110) was not large enough to ensure adequate power.

Taken together, our results mirror and extend the findings of previous studies and provide additional evidence that critical weight loss (≥4.6 %) was an independent prognostic factor for OS, FFS, and LR-FFS, irrespective of chemotherapy modality, radiotherapy technique, and BMI. The underlying reason may mainly involve malnutrition. Previous studies showed patients presenting with malnutrition experience more unplanned treatment delays or interruptions and poor overall survival in HNC patients [16, 22, 23]. Conversely, well-nourished patients can tolerate treatment better with fewer complications, recover faster after treatment and maximize quality of life [24–27]. In addition, during curative treatment (especially concurrent chemoradiotherapy) in NPC patients, the majority of patients present treatment-related toxicities, of which dysphagia caused by acute mucositis is one of the most prominent [28]. These acute toxicities bring to discomfort and difficulties with eating. Then insufficient food intake in the malnourished patients impaired the immune system [29], which further compromised the effect of radiotherapy on localregional control [30–32]. Moreover, it has been confirmed that that a severe deficiency of peripheral blood iNKT cells in patients with head and neck cancer was significantly related to poor clinical outcome [33]. Langius et al. [6] found that patients with CWL had significantly lower numbers of T cells and more often a low iNKT cell level compared with patients without CWL. In brief, weight loss is one of the main symptoms of malnutrition, which further cause immune suppression.

In this study a significant and independent impact of CWL on long-term survival of nasopharyngeal carcinoma patients was established. Thus clinical intervention to prevent therapy-associated weight loss was warranted. Clinical guidelines recommend enteral nutrition should be started if undernutrition already exists or if food intake is markedly reduced for more than 7–10 days [34], but both the enteral feeding methods and what the supplemental formula should contain are still debated [35, 36]. Further studies will be needed to address the research gaps in NPC.

Our study has several strengths. Firstly, we performed multivariate analyses adjusted for age, gender, T stage, N stage, treatment group, BMI, smoking status, radiotherapy dose, while most of previous studies analyzed the effect of CWL on the prognostic without adequate adjustment for relevant prognostic factors, thus significant differences were covered by other confounding variables. Secondly, two subsets were developed to confirm the significant differences. Lastly, we further carried out propensity score matching in two subsets to adjust for differences in baseline data. Based on these observations, we feel confident in our results.

Still, the limitations of our study are related to its retrospective nature and the data were obtained exclusively at one center. Next, Comorbidities like cardiovascular diseases, diabetes significantly affect prognosis of NPC patients [37], and further exacerbate malnutrition. However comorbidities appear to be more common in elderly patients, the percentage of elderly patients (≥65) in our study are relatively small (7.9 %), thus the potential confounding effect of comorbidities is not the main aspect. Thirdly, the sample size (*n* = 110) in IMRT cohort is not large enough to ensure adequate power.

## Conclusions

In summary, our data suggest that critical weight loss has a significant and independent impact on long-term survival in nasopharyngeal carcinoma patients. There is a clear distinction between patients with and without CWL. This emphasizes the importance of identification and optimal treatment of weight loss during NPC treatment in future.

## Additional file

Additional file 1: Figure S1. Comparison of overall survival among patients with ≥5 % weight loss, patients with <5 % weight loss, and patients with weight gain and without weight loss. (TIF 34 kb)

## Abbreviations

3D-CRT: three-dimensional conformal radiotherapy
AJCC: American Joint Committee on Cancer
BMI: body mass index
CRT: combined chemo-radiotherapy
CWL: critical weight loss.
D-FFS: distant failure-free survival rates
ECT: emission computed tomography
FFS: failure-free survival rates
HNC: head and neck cancer
HR: hazard ratio
IMRT: intensity-modulated radiotherapy
KPS: Karnofsky performance scale
LR-FFS: locoregional failure-free survival rates
NPC: nasopharyngeal carcinoma
OS: overall survival rates
ROC: receiver operating characteristic curve
RT: radiotherapy
UICC: Union for International Cancer Control
WL: body weight loss
