# Supplementary figures and images for: Critical weight loss predicts poor prognosis in nasopharyngeal carcinoma
Source: BMC Cancer. 2016 Feb 29;16:169. doi: 10.1186/s12885-016-2214-4 (PMC4772285; doi:10.1186/s12885-016-2214-4)

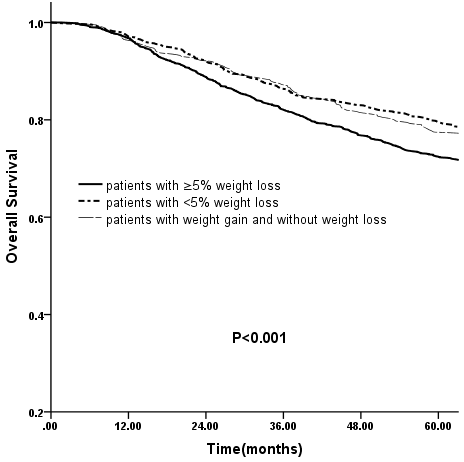

Supplement: Additional file 1: Figure S1. — Comparison of overall survival among patients with ≥5 % weight loss, patients with <5 % weight loss, and patients with weight gain and without weight loss. (TIF 34 kb) [file 12885_2016_2214_MOESM1_ESM.tif]
